# Resistance Training Volume Load with and without Exercise Displacement

**DOI:** 10.3390/sports6040137

**Published:** 2018-11-03

**Authors:** W. Guy Hornsby, Jeremy A. Gentles, Paul Comfort, Timothy J. Suchomel, Satoshi Mizuguchi, Michael H. Stone

**Affiliations:** 1 Athletic Coaching Education, College of Physical Activity and Sport Sciences, West Virginia University, Morgantown, WV 26505, USA; 2 Center of Excellence for Sport Science and Coach Education, Department of Sport, Exercise, Recreation and Kinesiology, East Tennessee State University, Johnson City, TN 37614, USA; GENTLESJ@mail.etsu.edu (J.A.G.); harahara10@hotmail.com (S.M.); STONEM@mail.etsu.edu (M.H.S.); 3 Directorate of Sport, Exercise and Physiotherapy, University of Salford, Salford, Greater Manchester M5 4WT, UK; p.comfort@salford.ac.uk; 4 Department of Human Movement Sciences, Carroll University, Waukesha, WI 53186, USA; timothy.suchomel@gmail.com

**Keywords:** volume load, athlete monitoring, exercise displacement, resistance training

## Abstract

Monitoring the resistance training volume load (VL) (sets × reps × load) is essential to managing resistance training and the recovery–adaptation process. Subjects: Eight trained weightlifters, seven of which were at national level, participated in the study. Methods: VL was measured both with (VLwD) and without (VL) the inclusion of barbell displacement, across twenty weeks of training, in order to allow for comparisons to be made of these VL calculating methods. This consisted of recording the load, repetition count, and barbell displacement for every set executed. Comparisons were made between VL and VLwD for individual blocks of training, select training weeks, and select training days. Results: Strong, statistically significant correlations (r ≥ 0.78, *p* < 0.001) were observed between VL and VLwD between all training periods analyzed. *t*-tests revealed statistically significant (*p* ≤ 0.018) differences between VL and VLwD in four of the seven training periods analyzed. Conclusion: The very strong relationship between VL and VLwD suggest that a coach with time constraints and a large number of athletes can potentially spare the addition of displacement. However, differences in percent change indicate that coaches with ample time should include displacement in VL calculations, in an effort to acquire more precise workload totals.

## 1. Introduction

Appropriate quantification of resistance training volume is believed to be a necessary step in understanding the link between training and the associated adaptations. Detailed resistance training studies report estimates of work from all resistance training sessions, allowing their studies to be reproducible [1,2,3,4], and coaches commonly track the volume of their exercise prescriptions. Acute and prolonged physiological responses to stress derived from resistance training can include hormonal alterations [5,6], increased energy expenditure [7], and neuromuscular fatigue [8]. Häkkinen [8] demonstrated that the greater the amount of resistance training work performed during a single training session, the greater the physiological disturbance [8]. Thus, more accurate estimations of work (training volume) could be beneficial in associating training volume with potential fatigue and recovery resulting from the disturbance of homeostasis. Monitoring resistance training volume can be critical for several reasons:

1. It allows coaches to monitor whether or not the pre-planned training volume closely matches the volume actually performed in training;
2. It allows coaches to better achieve targeted volume ranges for specific phases of training that involve a targeted objective (e.g., a strength endurance phase requires a greater training volume than a power phase);
3. It allows superior management of training volume from one phase (e.g., block) of training to the next (e.g., a desired drop in training volume);
4. It allows long term monitoring (e.g., when an athlete returns to a similar block of training, are they performing more work?).

Exercise displacement is calculated by the distance covered for a given movement task, and for free weight barbell exercises, displacement can be assessed as the distance the barbell travels during the concentric (vertical) portion of a resistance exercise for a single repetition. In a review on quantifying workloads in resistance training, Haff [9] explains that the mechanical work performed by an athlete can be quantified by multiplying the athlete’s force times displacement for each repetition. From a study by McBride et al. [10], in which four different volume assessment protocols were compared using force plate assessments, the authors stated that “total work” (TW) (force (N) × displacement (m)) was the “most valid” of the four resistance training quantification protocols examined. This was based upon TW being a direct measure of mechanical work and the other three volume estimations (reps × load, time under tension, and repetitions × (body mass − shank mass + external load)) each having specific limitations, as noted by the investigators [10]. 

Volume load (VL) (repetitions × load) has been reported in long-term resistance training studies for the past several decades. Haff [9] explains that displacement can be added to VL, the most common resistance training volume calculation, thus resulting in VLwD (repetitions × load × displacement). This may be warranted, because, for a practitioner, measuring the forces generated for every repetition is impractical. In 1987, Stone et al. [11] published a descriptive study on high repetitions (sets of 10) of back squats and an individual’s physiological response. To the authors knowledge this study was the first to include displacement in VL calculations. Over the last several years, Stone and colleagues have begun to use VLwD as an estimate of work in resistance training studies, for example, Bazyler et al. 2017 [4], Bayzler et al. 2018 [12], Caroll et al. [13], and Hornsby et al. [14]. 

Over the course of a macrocycle, exercises can change based on the overall goals of a specific block of training. Thus, the tracking of training volume from one phase of training to the next is a common consideration for the management of resistance volume. Block periodization models commonly involve a transition across the macrocycle from periods of higher volume to periods of lower volume. Though changes in the number of overall repetitions plays a major role in resultant volume alterations (e.g., decreasing across a macrocycle), so too does the manipulation of training load, as well barbell displacement alterations, via changes in exercises. Exercises commonly change from one phase (block) to the next due to changes in the coaches’ adaption objective(s). For example, during the accumulation block, training may consist of a greater amount of large range of motion exercises, whereas during a realization block, with a taper, a greater amount of partial range of motion exercises may be included in the training program. Exercise intensity (i.e., external load for a given resistance exercise) is commonly factored into volume estimations (e.g., VL), and its impact on an individual’s acute physiological response (e.g., heavier or lighter loads) has been well examined [5]. 

McBride et al.’s [10] study demonstrated that exercise displacement provides a more accurate assessment of resistance training volume. However, McBride et al. [10] calculated exercise displacement along with force output and did not compare displacement to VL. Previously, no longitudinal study has been performed comparing changes in VL with and without exercise displacement. Practitioners are likely less interested in the accuracy of their resistance training volume assessment compared to how well changes in their volume calculation reflect changes in an athlete’s overall workload. Based on Haff [9] and McBride et al. [10], the inclusion of exercise displacement into VL provides a more accurate estimation of training volume. The authors of the present study were interested in how worthwhile it would be, for the coach or strength and conditioning specialist, to include displacement measures along with VL calculations; specifically, is it worth the additional time to collect? If differences exist between VL and VLwD it can be surmised that VLwD is a more accurate estimation of work performed. Thus, the aim of this study was to compare the VL and VLwD during individual blocks of training, select training weeks, and select training days to determine if there are meaningful differences between these methods. For example, if differences exist between VL and VLwD when changing from one specific training period to another, this would suggest that VLwD is worth the additional time. The authors hypothesized that the inclusion of barbell displacement would, for certain periods of training (e.g., when greater changes in volume occur), result in differences in reported workloads performed by the weightlifters. The authors considered it advantageous to observe training in athletes in an ecologically valid environment (i.e., normal training and coaching conditions) over a prolonged period, as this better mirrors real-world training situations. 

## 2. Materials and Methods

### 2.1. Experimental Approach to the Problem

All of the training data was retrospective information collected, over the course of twenty weeks, by weightlifting coaches and sport scientists of the East Tennessee State University Designated Olympic Training Site as part of an ongoing athlete monitoring program. The training took place in the Exercise and Sport Science Laboratory weight room on the campus of East Tennessee State University. Data collected was approved to be utilized for the purposes of this study by East Tennessee State University’s Institutional Review Board.

Of particular interest was the comparison between various training phases (e.g., training blocks), training weeks (microcycles), and individual training days (intra-microcycles), and specifically for situations in which greater contrasts existed in terms of repetitions and/or training intensity. For example, sets of 10 repetitions compared to sets of five repetitions, or a heavier training day (90%–95%) compared to a lighter training day (70%–75%). 

#### 2.1.1. Athletes

Data from eight well-trained weightlifters (Table 1) were utilized in the study, across five months of training. Of the eight, seven were national level weightlifters (three U.S. National Championship qualifiers, one American Open qualifier, three National Collegiate Championship qualifiers) and one was a regional level weightlifter. Most likely due to the lifters being experienced, little variation existed in displacement within a particular exercise for the same weightlifter (SD < 0.05 m). The variability in body size (height = 174 ± 8.4 cm, body mass = 88.4 ± 22.7 kg) were of important consideration as this heavily influences the exercise displacements. 

#### 2.1.2. Procedures

The monitoring of VL across six phases consisted of recording the load and repetition count, whilst factoring in barbell displacement, during the concentric portion of the exercise. Displacement for each exercise was measured using the V-scope 120 (Lipman Electronic Engineering Ltd., Ramat Hahayal, Israel). Created for real time bar path analysis for weightlifters, the V-scope involves placing a cap on the end of a weightlifting barbell that contains an ultrasound emitting device. Three infra-red emitting towers, interfaced with a computer, detect the ultrasound beam. Through a triangulation method, accurate measurements of displacement and barbell path can be made [14,15].

V-scope methods utilized were based on Stone et al. [15]. Displacement measurements took place before the five months of training began, and the mean was taken from three trials for each exercise for each weightlifter. High intra-class correlations coefficients (ICCs > 0.9) were displayed for displacement during each exercise. Reliability and validity of the V-scope (frequency: 66 Hz) was assessed by moving the V-scope cap by hand, vertically, along a straight edge, across a pre-determined distance (50 cm). The total displacement difference between trials was less than 1 cm (<1%), and high ICCs were obtained (>0.99). Exercises involving two concentric portions involved measuring segments of the full movement and adding the segments together (e.g., clean = clean pull into the catch position + front squat out of the catch). 

Mathematically, calculating VL times displacement (VLwD) entailed multiplying the displacement for the given exercise by the number of repetitions and the given load for each repetition (sets × repetitions × load × displacement). 

### 2.2. Training Prescription

**Figure 1:**
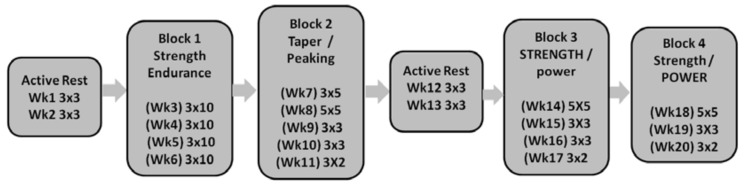
Sets and repetitions with corresponding training foci across the 20 weeks.

The training prescription involved six distinct training blocks (active rest > strength endurance > taper > active rest > STRENGTH/power > strength/POWER) and was based on scientific literature and common training approaches in weightlifting [13,14,16,17]. Variation is a key component of periodized training prescription, which allows a coach to manage and guide training variables toward a specific adaptation goal [13,14]. Figure 1 displays the set and repetition scheme executed by the weightlifters, and demonstrates that different training blocks involved different set and repetition prescription. Table 2 displays the relative intensities for the 20 weeks of training. Relative intensities (e.g., percentage of set-rep best) allow for proper fatigue management and, along with the set and repetition scheme and the exercises, dictate the resistance training volume [14,17]. 

Table 3 displays all of the exercises the weightlifters performed during the 20 weeks, and Table 4 displays the average displacements for each exercise, ordered from largest to smallest displacement. These exercises are commonly used in training prescriptions for weightlifters [14], and have been used successfully for athletes of other sports as well [4,12]. Though barbell displacements are certainly specific to an individual athlete, this order of displacements is likely to be found for other athletes due to the common demands of a given exercise. For example, a snatch requires moving the bar farther than a snatch pull from the floor, regardless of the height and limb lengths of the athlete. 

### 2.3. Statistical Analyses

Statistical analyses involved the comparison of VL and VLwD (SPSS statistical analysis software version 19, Armonk, NY, USA), which was used to determine Pearson correlation coefficients (r = −1.0 to 1) to identify relationships between training blocks, as well as select training weeks and select training days [17]. Correlations were evaluated as follows: Small (0.1), moderate (0.3), and large (0.5). A test of multi-collinearity [18] was performed (VL vs. VLwD) displaying a variance inflation factor (VIF) of 1.0. *t*-tests (*p* < 0.05) were used to analyze the changes (percent differences) from one training block to the next, as well as the changes between two selected training weeks and within a selected training week. An a priori alpha level was set at *p* ≤ 0.05. Cohen’s *d* effect sizes were also calculated to determine the magnitude of any observed differences, and classified as trivial (< 0.20), small (0.20–0.59), moderate (0.60–1.19), large (1.20–1.99). and very large (≥2.0) [19].

## 3. Results

### 3.1. Relationships

Volume load (VL) correlated strongly with VLwD for all training phases, weeks, and days analyzed (Table 5). All of the correlations were large and significant (r > 0.78, *p* < 0.001).

### 3.2. Percent Change

Percent change data displayed two key findings. Percent change for both VL and VLwD appeared to demonstrate the same general direction of change in resistance training work for various periods of training (Table 6). However, the results of the paired samples *t*-tests demonstrated that the percent change between VL and VlwD was statistically different in four of the seven periods analyzed (Table 6). Additionally, six of the seven periods displayed a strong effect size (Table 6). 

### 3.3. Visual Representation

**Figure 2:**
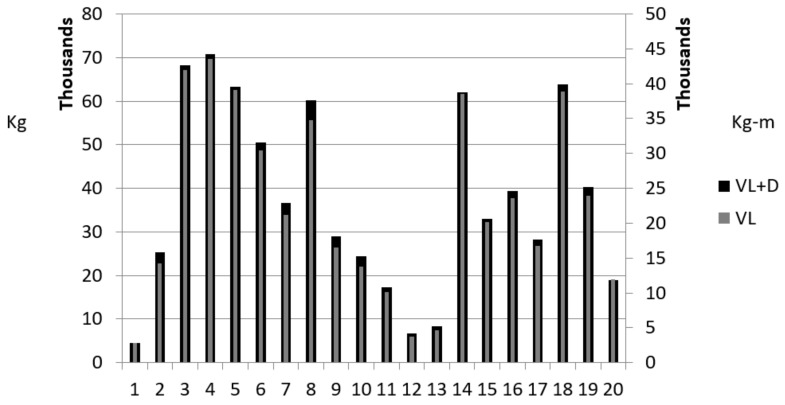
VL and VLwD changes across the 20 Weeks.

Coaches often “track” resistance training volumes via graphical representations [14,20]. Comparable to the percent change data, Figure 2 demonstrates that fluctuations for VL and VLwD were similar. Simply put, when VL rose, VLwD rose, and when VL decreased, so too did VLwD. However, the amount of space between VL and VLwD did fluctuate from week to week.

## 4. Discussion

Good fatigue management is paramount. Importantly, the percent changes for four of the seven time periods investigated demonstrated statistically significant differences. These differences were displayed when a comparison was made between high(er) and low(er) volume training blocks, in which there was a large contrast in training volume. Similar results were noted for heavy and light days. Thus, if a coach wants to be assured that changes in training volume are best represented it is worth including displacement (VLwD). 

**Figure 3:**
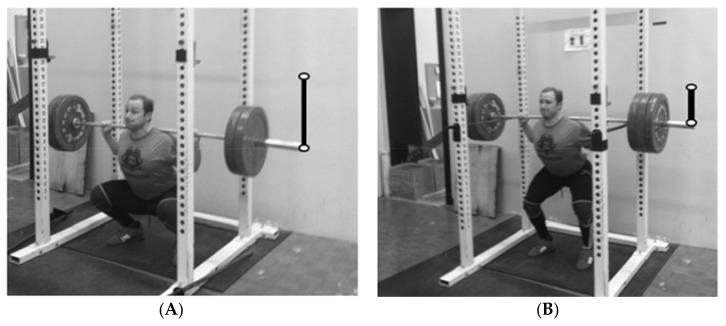
Exercise displacement comparison. (**A**) Weightlifter executing a full squat; and (**B**) weightlifter executing a quarter squat.

In addition to disparities in volume as a result of changes in the number of prescribed repetitions, another important consideration for changes in VLwD, and not necessarily VL, is changes in range of motion (displacement) due to changes in exercise selection from one training block to the next. For example, full movements may eventually switch to partial movements later in the macrocycle. A switch from a full movement exercise to a partial movement exercise (e.g., full squat replaced with a quarter squat) is common when a coach implements either a taper or an in-season maintenance program. Heavier loading is typically used with partial movements (but less displacement) and thus, when comparing a quarter squat (see Figure 3 below) to a full squat, the load for the quarter squat will be much higher for the same relative training intensity. However, when taking into account barbell displacement, the VL is less than when compared to executing the full movement. Table 7 illustrates this difference. Additionally, based on the data generated from the current study, differences in relative work when switching from block to block can be underestimated using only VL (Table 7).

Although the use of VLwD requires some additional effort, its use provides a more accurate characterization of loading. Based on the data in the present study, a coach may mischaracterize the changes between loading periods by using VL. Factors influencing a potential difference between a change in VL and VLwD likely include the changes in the number of partial versus full movements, and the relative alterations between loading periods (e.g., heavy and light days, high volume block to low volume block). 

## 5. Conclusions

The direct measurement of weight training volume can involve measuring forces, displacement of the external load, and the energy expenditure. Thus, researchers can examine various methods of estimating weight training volume by comparing it to direct measurements. Volume load has been shown to be a reasonable estimate of work when compared to the direct measurements of forces and barbell displacement (i.e., mechanical work) in the back squat; however, is not as accurate as when including exercise displacement (one of the two variables for quantifying mechanical work) [10]. 

The process of calculating VLwD is certainly more time consuming than for VL; however, sound planning and structure can minimize the burden and provide a rather efficient system. This can include: (1) Measuring exercise displacements before implementing several blocks of training (e.g., the first day of team training for a given year or season); and (2) inserting the displacements for the given exercises into excel, allowing VLwD to be “auto-calculated”, following data imputation (e.g., loads and repetitions). Based on the present study findings and previous research [10], despite the calculation VLwD being more time-consuming compared to VL (i.e., load x repetitions), coaches and sports scientists are recommended to use VLwD to provide estimates of work in a more meaningful fashion.

## Figures and Tables

**Table 1:** Descriptive weightlifters’ data.

Sex	N	Height (cm)	Body Mass (kg)	RT Age (years)	WL Age (years)	Snatch (kg)	Clean and Jerk (kg)
Males	5	178 ± 6.9	103.1 ± 13.7	9.8 ± 5.1	5.1 ± 5.0	107.4 ± 27.6	135 ± 32.9
Females	3	166.2 ± 4.6	64.8 ± 2.9	5.3 ± 2.5	3.0 ± 1.4	55.3 ± 6.4	69 ± 8.5

RT = resistance training, WL = weightlifting.

**Table 2:** Percentage of set-rep best across the 20 weeks of training.

Week	Monday	Wednesday	Thursday	Friday	Saturday
1		60–65%		60–65%	
2	60–65%	65–70%		65–70%	
3	75–80%	70–75%	80–85%		80–85%
4	80–85%	70–75%	80–85%		85–90%
5	85–90%	70–75%	75–80%		90–95%
6	75–80%	70–75%	75–80%		≥95%
7	75–80%	70–75%	80–85%		80–85%
8	80–85%	75–80%	80–85%		85–90%
9	85–90%	75–80%	75–80%		90–95%
10	90–95%	80–85%	70–75%		90–95%
11	75–80%	70–75%	75–80%		≥95%
12	70–75%	70–75%		75–80%	
13	75–80%	70–75%		70–75%	
14	75–80%	70–75%	80–85%		80–85%
15	80–85%	75–80%	80–85%		85–90%
16	85–90%	75–80%	75–80%		90–95%
17	90–95%	80–85%	70–75%		90–95%
18	75–80%	75–80%	80–85%		80–85%
19	80–85%	75–80%	80–85%		85–90%
20	85–90%	80–85%	70–75%		≥95%

Note: Tuesdays and Sundays were always rest days.

**Table 3:** Exercises for each block of training.

Block 1: Weeks 3–6	Block 2: Weeks 7–11	Block 3: Weeks 14–17	Block 4: Weeks 18–20
Monday/Thursday	Monday/Thursday	Monday/Thursday	Monday/Thursday
AM	AM	AM	AM
Squats	Squats (drop after 2nd week)	Squats	Squats
PM	PM	PM	PM
Front squats	Push press	Push press	Push jerks (front squat 1st rep)
Standing press	Change to push jerks on week 3	Jerk recoveries	Jerk recoveries
Wednesday	Wednesday	Wednesday	Wednesday
AM	AM	AM	AM
CGSS	CGSS	CGSS	CGSS
CGMTP	CG pulls—floor	CG pulls—floor	CG pulls—floor
PM	PM	PM	PM
CGSS (20% less)	CGSS (20% less)	CGSS (20% less)	CGSS (20% less)
CG pulls—knee	CG pulls—knee	CG pulls—knee	CG pulls—knee
CGMTP	CGMTP	CGMTP	CGMTP
SLDL	SLDL	SLDL	SLDL
Saturday	Saturday	Saturday	Saturday
SGSS	SGSS	SGSS	SGSS
Undulating snatch 10 × 1	Undulating snatch 5 × 1	Undulating snatch 5 × 1	Undulating snatch 5 × 1
(up to 85% of best on week 4)	(up to 90% of best on week 4)	(up to 85% of best on week 4)	(up to 90% of best on week 2)
SG–SLDL	Undulating clean and jerk 5 × 1	Undulating clean and jerk 5 × 1	Undulating clean and jerk 5 × 1
Lateral raises	(up to 90% of best on week 3)	(up to 80% of best on week 3)	(up to 90% on week 1)
	SG–SLDL	SG–SLDL	SG–SLDL

SG: Snatch grip; CG: Clean grip; CGSS: Clean grip shoulder shrugs; CGMTP: Clean grip mid-thigh pull; SLDL: Stiff-legged deadlifts; SGSS: snatch grip shoulder shrugs.

**Table 4:** The exercises executed during the weightlifters’ 20 weeks of training and the corresponding displacements.

Exercise	Mean Displacement (m)
Snatch	2.21 (±0.12)
Clean	1.88 (±0.08)
Power snatch	1.53 (±0.14)
Snatch grip pull from floor	1.08 (±0.10)
Clean grip pull from floor	1.01 (±0.07)
Push press	0.76 (±0.10)
Back squat	0.71 (±0.04)
Push jerk	0.70 (±0.10)
Jerk	0.68 (±0.10)
Front squat	0.68 (±0.06)
Overhead squat	0.67 (±0.05)
Clean grip pull from knee	0.67 (±0.08)
Snatch grip SLDL	0.58 (±0.07)
Press	0.56 (±0.05)
Clean grip SLDL	0.55 (±0.06)
Behind Neck Press	0.53 (±0.05)
Mid-thigh pull—Snatch grip	0.47 (±0.04)
Mid-thigh pull—Clean grip	0.39 (±0.05)
Lockouts	0.07 (± 0.01)

Note: Exercises involving two concentric portions required the full movement segments to be measured, and then the segments were added together (e.g., snatch = snatch pull + overhead squat).

**Table 5:** Comparison between volume load (VL) and volume load with displacement (VLwD) for various portions of the training prescription.

Description of Training Period	Pearson’s r
Active rest one—2 weeks	0.99
Strength endurance—4 weeks	0.98
Taper—5 weeks	0.99
Active rest two—2 weeks	0.94
STRENGTH/power—4 weeks	0.98
strength/POWER—3 weeks	0.99
Week of 3 × 10	0.96
Week of 3 × 5	0.93
Training day—3 × 5, 90%–95%	0.96
Training day—3 × 5, 70%–75%	0.78

**Table 6:** Comparison between VL and VLwD.

Description of Training Period Change	Measurement	% Δ AVG.	*p* Value	Cohen’s *d*
Active rest to strength endurance	VL	1303.1 (±495.0)	0.002	0.63
	VLwD	1018.0 (±400.1)		
Strength endurance to taper	VL	−38.5 (±5.0)	0.00	2.45
	VLwD	−27.9 (±3.6)		
Taper to active rest	VL	−91.8 (±2.3)	0.105	0.27
	VLwD	−91.2 (±2.1)		
Active rest to strength/POWER	VL	1181.8 (±307.7)	0.003	0.93
	VLwD	929.2 (±231.1)		
STRENGTH/power to strength/POWER	VL	−20 (±11.4)	0.613	0.06
	VLwD	−20.6 (±10.4)		
Week of 3 × 10 to week of 3 × 5	VL	−30.2 (±10.1)	0.018	0.8
	VLwD	−21.1 (±12.2)		
Heavier training day (90%–95%) to lighter training day (70%–75%)	VL	−32.9 (±18.3)	0.089	0.77
	VLwD	−21.5 (±10.3)		

**Table 7:** Example: Volume load and training intensity (average load) comparison of the full squat and quarter squat.

Sets and Reps	L	VL	TI	D	VLwD
Full squat					
1 × 10	60	600	60	0.55	330
1 × 10	100	1000	100	0.55	550
3 × 10	130	3900	130	0.55	2145
Total (50 reps)		5500			3025
Mean reps per set (10)	110		110	0.55	
Quarter squat	L	VL	TI	D	VLwD
1 × 10	60	600	60	0.28	168
1 × 10	150	1500	100	0.28	420
3 × 10	200	6000	130	0.28	1680
Total (50 reps)		8100			2268
Mean reps per set (10)	162		162	0.28	

Note: In this scenario the VL for quarter squats provide an inflated estimate of work. Thus, if comparisons are being made between the two exercise sessions, VLwD can provide a more accurate estimate of the work performed and the physiological impact. L: Load (kg); VL: Volume load (sets × repetitions × load); TI: Training intensity (VL/reps (kg)); D: Bar displacement (or weight displacement) in meters; VLwD: VL × displacement (kg).
